# Correlations Preceding High-Intensity Events in the Chaotic Dynamics of a Raman Fiber Laser

**DOI:** 10.3390/e21020151

**Published:** 2019-02-05

**Authors:** Andrés Aragoneses, Yingqi Ding

**Affiliations:** 1 Department of Physics, Eastern Washington University, Cheney, WA 99004, USA; 2 Department of Physics and Astronomy, Carleton College, Northfield, MN 55057, USA

**Keywords:** forecasting, complex dynamics, fiber laser, chaos, ordinal patterns

## Abstract

We study the time series of the output intensity of a Raman fiber laser with an ordinal patterns analysis in the laminar-turbulent transition. We look for signatures among consecutive events that indicate when the system changes from triggering low-intensity to high-intensity events. We set two thresholds, a low one and a high one, to distinguish between low intensity versus high-intensity events. We find that when the time series is performing low-intensity events (below the low threshold), it shows some preferred temporal patterns before triggering high-intensity events (above a high threshold). The preferred temporal patterns remain the same all through the pump current range studied, even though two clearly different dynamical regimes are covered (laminar regime for low pump currents and turbulent regime for high pump currents). We also find that the turbulent regime shows clearer signatures of determinism than the laminar regime.

## 1. Introduction

Besides the innumerable technological applications, lasers are a fascinating tool for the study and characterization of nonlinear dynamics in a laboratory-controlled manner [1]. Being able to generate and control complex dynamics with lasers has been important for studying and understanding chaos [2,3], stochastic and coherent resonance [4,5], rogue waves [6], for generating encrypted communications with chaos [7], for developing subwavelength position sensing protocols [8], or developing neuro-inspired optical devices for computation [9,10,11].

One particular case of a laser manifesting complex dynamics is the Raman fiber lasers with optical feedback [12]. The light amplification of these lasers involves nonlinear interactions of millions of longitudinal cavity modes. This leads to a highly complex dynamics in the output intensity of the laser. In previous studies [13,14], it was found that behind the high complexity of the time series of the output intensity of the fiber laser, the system showed some structure that could be unveiled using an ordinal patterns analysis. It was found that the laser executes some preferred intensity and temporal patterns, and those patterns helped to detect a laminar to turbulent transition as the pump power of the laser was increased [12,13]. In [14], through a simple mathematical model and an ordinal patterns analysis, it was also seen that the transition was related to internal frequencies of the dynamics.

In this paper, we use the ordinal patterns analysis to study the complex behavior of the output intensity of the fiber laser, to forecast when the laser is changing from emitting low-intensity light to emit high-intensity light. The ordinal patterns analysis allows finding temporal or intensity correlations in a highly complex time series. These correlations reveal memory signatures in the dynamics of the system that can be related to the structure of the phase space. Our goal is to characterize the changes in the magnitude of the events to be able to statistically forecast when low magnitude events trigger high magnitude events.

Forecasting high magnitude or extreme events is of great importance in many fields, from brain behavior to earthquakes, electronic circuits, or stock markets [15,16,17,18,19]. Because each dynamical system has intrinsic features, different statistical tools have to be used for each system. In [20], the authors used the ordinal patterns analysis to forecast extreme events in the time series generated with the Lang–Kobayashi [21,22] model to simulate a diode laser with current injection. They analyzed intensity correlations and found that some preferred patterns took place before the system triggered one extreme event. In [23], the authors analyzed the experimental times series of a low-power diode laser submitted to optical feedback in the low-frequency fluctuations regime, close to the lasing threshold of the laser (below 1 mW). They analyzed the temporal correlations of the events and also found some preferred patterns happening before a change from low- to high-intensity events and from high- to the low-intensity events. In this later work, the preferred patterns were different from those in the former.

Our work extends the range of applicability of the method to high-power Raman fiber laser (powers around 1 W). We show that the method also unveils signatures in the complex dynamics of the system under study. We find preferred patterns that precede high-intensity events. The fact that the likelihood of some patterns reaches 70% in some cases, while only about 10% in other cases, suggests a very deterministic behavior before a high-intensity event is triggered. These patterns can be helpful in forecasting events in the complex dynamics of a Raman fiber laser in the laminar and turbulent regimes.

## 2. Forecasting Method

The ordinal patterns analysis method (OPAM) was introduced by Bandt and Pompe in 2012 [24] and studies correlations between consecutive events. It allows computing the permutation entropy of the dynamical system by calculating the probabilities of different patterns in the time series. It defines the patterns by comparing the values of *n* consecutive events in a time series (either magnitudes or time intervals between events) and computes the probabilities of each of the n! possible patterns. The permutation entropy reveals information on the complexity of each time series, but studying the probabilities of the different ordinal patterns allows us to extract information about the underlying structure of the dynamics. For a purely-stochastic process, where no correlations exist between events, one would expect to find all patterns equally probable (P=1/n!), while for systems that present some intensity or temporal correlation, one would expect some structure in the probabilities of the patterns. This method has been used to characterize complex dynamics based on the patterns’ probabilities [25,26,27,28], to distinguish between stochasticity and determinism [29], or to identify transitions in the dynamics of complex networks [30]. In a recent paper [23], the method was used to classify events into two types and to forecast when the system decides to change from one type of event to the other one.

The OPAM transforms the original series of *m* events, $X=\{x_{1},x_{2},\ldots ,x_{i},\ldots \}$, into a series of m−n patterns. To do so, it considers *n* consecutive events, assigns them correlative integer numbers (0, 1, 2, ..., *n*), and then orders them from smaller to larger values of the event. For example, for dimension n=2, if $x_{i}<x_{i+1}$, it labels that pattern as 01; if $x_{i+1}<x_{i}$, it labels that pattern as 10. For dimension n=3, we have six possible patterns (012,021,102,120,201,210), i.e., if $x_{i}<x_{i+1}<x_{i+2}$, it labels that pattern as 012; $x_{i}<x_{i+2}<x_{i+1}$ is 021.

This can be applied to the actual values of the events ({x(i)}), the magnitudes in the time series, but it can also be applied to the time intervals between events ({t(i+1)−t(i)}). The former would give us information about intensity correlations among consecutive events, while the latter about temporal correlations.

For the fiber laser under study, both intensity and temporal correlations were found (see Figure 2a,b from [13]), where some patterns were more likely to happen in the time series. The fact that the OPs are not equally probable means that the dynamics is not compatible with a stochastic process, but it is clearly deterministic. The system showed a higher degree of determinism at the laminar-turbulent transition.

## 3. Results

We analyze the output intensity of a quasi-cw (continuous-wave) Raman fiber laser formed of 1 km of normal dispersion fiber. The laser is placed between two fiber Bragg gratings that act as cavity mirrors. Time series are recorded for 625μs, with $5\times 10^{7}$ points and a discretization time of ∆t = 12.5 ps. The dynamics of the output intensity is recorded for a range of pump powers between 0.80 W and 1.50 W (see [12] for details). To compare among the different pump powers, time series are subtracted the average and normalized to have zero mean and unit variance.

**Figure 1:**
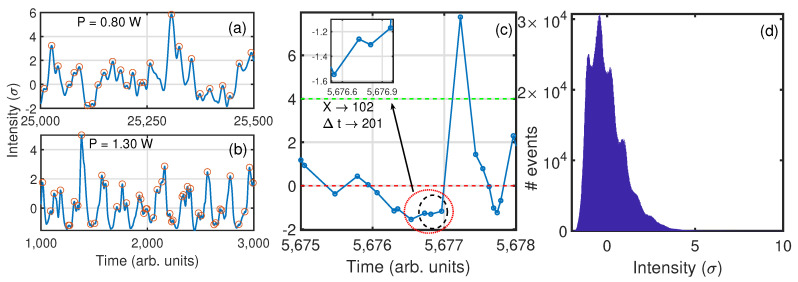
(**a**,**b**) Time series of the events (red circles) for two pump currents, one in the laminar regime, 0.80 W (**a**), and the other in the turbulent regime 1.30 W (**b**). The time series oscillate around zero, but present some intensity events larger than 4σ. (**c**) Detail of the time series where four low-intensity events are selected below $th_{0}=0$ (dashed red line) that precede a high-intensity event, above $th^{*}=4\sigma$ (dashed magenta line). The dotted red circle and the dashed black circle indicate the four (three) consecutive events below $th_{0}$ used to generate an ordinal pattern (OP). Three consecutive events are used when OPs are computed with intensities and four consecutive events when OPs are computed. (**d**) Histogram of events for a power of 1.20 W (in the turbulent regime). The distribution of events shows some structure and a tail that reaches values larger than 6σ.

Two different regimes can be distinguished in the output power of the Raman fiber laser as we vary the pump current. For lower values of pump power, the regime can be considered laminar, while for higher values of the pump current, it becomes turbulent (as was shown in [12]). The laminar-turbulent transition takes place at 0.90 W. The recorded intensity, I(t), for the different pump powers looks similar (see Figure 1a,b), despite it showing different coherence properties in each of the two regimes.

In order to compute the ordinal patterns, we focus our analysis on the peaks of the time series. By doing this, we are obtaining a new time series, in this case of events, where the events are the peaks of intensity.

Figure 1a,b shows a section of a time series of the output intensity of the laser for two different pump powers, one for the laminar regime (0.80 W) and the other for the turbulent regime (1.30 W). Peaks are indicated with red circles. These peaks will be considered as events in the ordinal patterns analysis. The time series of the output intensity show a wide distribution with most values around ±2σ and fewer events reaching values >6σ. Figure 1c shows four consecutive events and the corresponding OP generated. The black circle in Figure 1c indicates the three events considered to generate the OP with an intensity of the values ($X_{i+1}>X_{i}>X_{i+2}\to 102$), and the red circle indicates the four consecutive events considered to generate the OP with their inter-event time-intervals ($∆t_{i+1}<∆t_{i+2}<∆t_{i}\to 120$).

Figure 1d shows the histogram of the events for 1.00 W. This histogram shows a long tail for high intensities. This type of long-tail distribution is present in a wide variety of systems [15,16,17,18], which can lead to unwanted phenomena; hence the importance of understanding and being able to forecast when the system is going to perform events corresponding to the tail, sometimes referred to as extreme events.

Other statistical analysis such as kurtosis or skewness of the distribution of events could be performed, but they would not reveal relevant information about the temporal and intensity correlations among the events in the time series, and this is out of the scope of the present work.

We define a threshold, $th^{*}$, to classify the events as large when they are above that threshold. We are interested in predicting when the system changes from emitting low-intensity light (lower than $th^{*}$) to emitting high-intensity light (higher than $th^{*}$).

To forecast the change from low-intensity behavior to the high-intensity one, we impose that at least three consecutive events are below $th^{*}$ before the system goes to emit a high-intensity event. We compute the ordinal patterns (OPs) of the three inter-event time-intervals right before the system changes from low to high intensity. This will indicate if there are OPs that are more/less likely to happen before the transition, and which OPs these are.

**Figure 2:**
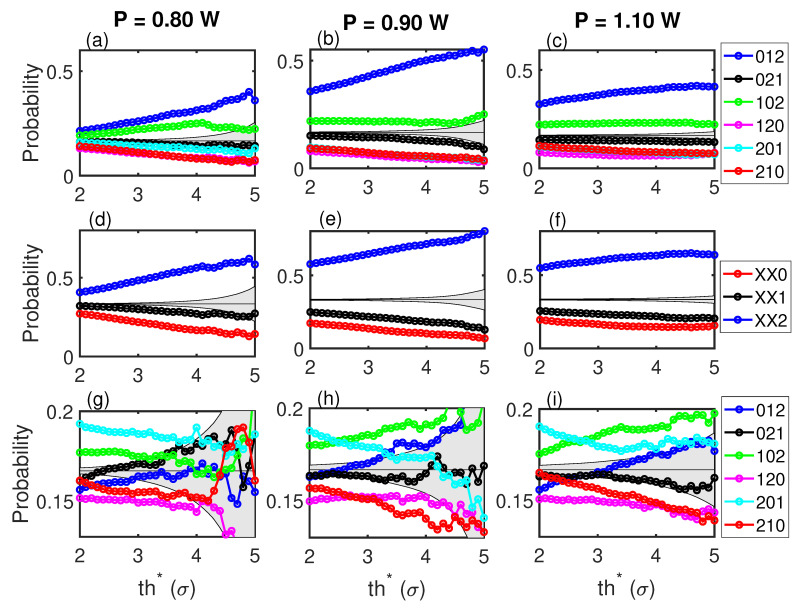
(**a**–**c**) Ordinal patterns computed with intensities. Three consecutive events below $th^{*}$ that precede an event above $th^{*}$ are considered for each OP. (**d**–**f**) The OPs are grouped into XX0=120+210, XX1=021+201, and XX2=012+102. This highlights the fact that the most likely OPs to happen before a jump from low-intensity events to high-intensity event are those where the last event is larger than the previous two. (**g**–**i**) OPs computed with inter-event time-intervals. The gray region corresponds to a stochastic process where all six patterns are equally probable.

Figure 2 shows the six OPs of dimension three versus the threshold, $th^{*}$, for three power outputs, P= 0.80 W (in the laminar regime), P= 0.90 W (at the transition), and P= 1.10 W (in the turbulent regime). The top row corresponds to the OPs generated with the intensity of the events. It can be observed that there are OPs that are more likely to happen and OPs that are less likely to happen, before the change from low-intensity to high-intensity events. This points to some structure in the complex dynamics that manifests as a preference of patterns before performing a high-intensity event. The gray region corresponds to the probabilities of a stochastic process of the six OPs equally probable. This region depends on the number of events in each threshold. For a high enough threshold, the number of high-intensity events preceded by low-intensity events is too low to have conclusive statistics, and therefore, the gray region widens, while the OPs probabilities lie in the gray region.

The second row in Figure 2 groups the six OPs into three, depending on the last event, to highlight this last event (the event preceding the high-intensity event) with respect to the previous two (Prob(XX0)=Prob(120)+Prob(210), the latter event is smaller than the previous two; Prob(XX1)=Prob(021)+Prob(201), the latter event is intermediate; Prob(XX2)=Prob(012)+Prob(102), the latter event is larger than the previous two).

After this, Figure 2d–f indicates that between 40% and 50% of the high-intensity events will be preceded by pattern XX2, i.e., of the three preceding events, the latest one is higher than the other two. In other words, this indicates that the change from low-intensity to high-intensity is not as sharp as it could be, as the peak preceding the high-intensity peak tends to be the highest of the three. This figure also shows that the OP probabilities do not depend on the threshold.

The bottom row of Figure 2 shows the OPs generated with inter-event time-intervals. For low thresholds, $th^{*}$, the probabilities are not compatible with a stochastic process, but they are much smaller than the ones computed with intensities. This shows that the dynamics presents less intense temporal correlations than correlations in magnitude. Furthermore, the hierarchy is different from the top to the bottom row. Furthermore, in a previous work [13], temporal correlations were found to be of less relevance than intensity correlations.

There is little effect of the power output of the laser on the probabilities of the OPs. There is a slightly higher probability right at the transition, but the hierarchies remain with the pump current.

**Figure 3:**
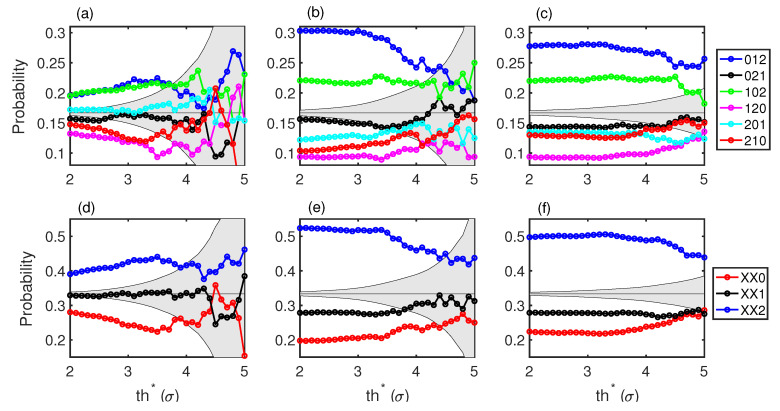
Ordinal patterns computed with intensities using two thresholds. A low threshold, $th_{0}=1\sigma$, imposes that the consecutive low events that form OPs are below $th_{0}$. A high threshold selects the high events that take place after the OP and are above $2\sigma \leq th^{*}\leq 5\sigma$. (**a**–**c**) correspond to the six OPs of dimension three. (**d**–**f**) correspond to the OPs combinations where the last of the three event is larger, in between, or shorter than the other two events. This more restrictive condition reduces the number of events and widens the gray area. As in Figure 2, the first row corresponds to 0.8 W, the middle row to 0.90 W, and the right row to 1.10 W.

Splitting the peaks into above and below a single threshold does not separate events into those that are much larger than average (events that can be considered as extreme events) and those events around and below average. In Figure 3, we consider two thresholds, a low one, $th_{0}$, and a high one, $th^{*}$ (in Figure 2c, two thresholds are indicated). We set the lower threshold at $th_{0}=1\sigma$ and scan the high threshold, $2\leq th^{*}\leq 4$. We then calculate the OP probabilities (see Figure 3).

We find that there are still preferred patterns before jumping from below $th_{0}$ to above $th^{*}$. The preferred OPs are the same as in Figure 2. This is interesting as the conditions to select the events to build the OPs are not the same. In the first case, it was less restrictive, and low events could be very close to $th^{*}$, while in the second case, all events that contribute to the OP are below $th_{0}$. The fact that the most probable forecasting OP is XX2 suggests that when the intensity of the low events grows to be near the low threshold, $th_{0}$, there is more chance for a high event to happen.

With this more restrictive condition of two thresholds, the number of events that satisfy that $x_{i-3}<th_{0}$, $x_{i-2}<th_{0}$, $x_{i-1}<th_{0}$, and $x_{i}>th^{*}$ is reduced. This is reflected in the wider gray regions in Figure 3 and in the fact that above $th^{*}=4\sigma$, the probabilities are compatible with a stochastic process, so we cannot use this as a forecasting method in that region. However, this is a characteristic of the dynamics of the system, as the high-intensity events are not rare and the system does not show too many consecutive low-intensity peaks before preforming a high-intensity peak. In any case, for power outputs of the laser above the transition (P≥ 0.90 W) and events larger than 3.5σ, we can still see that 50% of these intense events happen after the XX2 pattern.

Comparing these patterns probabilities with those of [23] where a similar analysis was performed, we find that the most probable OPs do not coincide; each system has different preferences.

**Figure 4:**
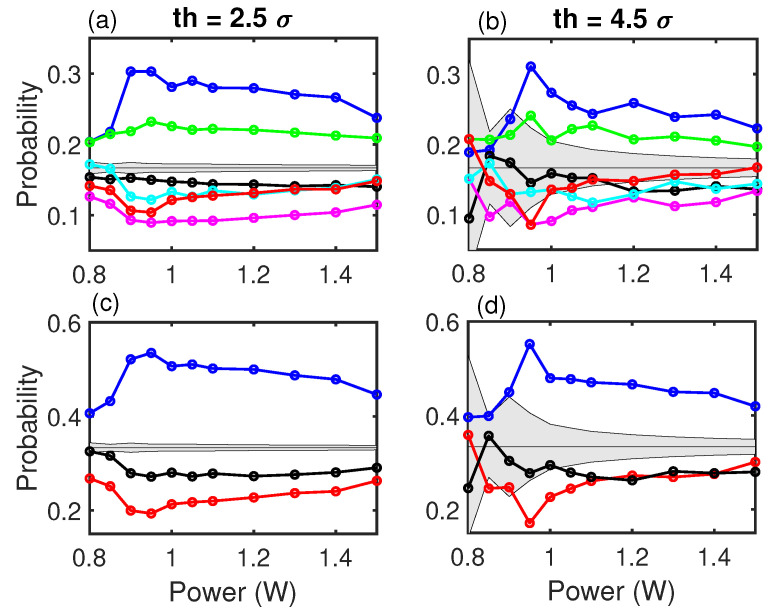
Probabilities of the ordinal patterns used to forecast versus pump power of the laser. (**a**,**b**) show the probabilities for the six OPs of dimension three. (**c**,**d**) show the probabilities for the grouped OPs. A clear qualitative change is seen at the laminar-turbulent transition. The forecasting signatures are clearer in the turbulent regime than in the laminar regime. In (**a**,**c**), the high threshold is 2.5σ, while in (**b**,**d**), the high threshold is 4.5σ.

Figure 4 shows the probabilities of the OPs with the forecasting condition (three consecutive events below the low threshold and the following event above the high threshold) for two different high thresholds, 2.5σ and 4.5σ. At the laminar-turbulent transition (0.90 W), there is a clear increase in the dispersion of the probabilities, indicating a change of forecasting signature. The dynamics of the system are more predictable in the turbulent regime than in the laminar regime. The dynamics in the laminar regime is closer to a random process than it is in the turbulent regime.

While the hierarchy of the OPs remains through the whole power range and is far from the gray region for th=2.5σ, for th=4.5σ, the available statistics places the laminar regime as compatible with a stochastic process, but the turbulent regime remains outside this gray region and shows the same probabilities (about 50% for the XX2 word).

## 4. Conclusions

We have studied the dynamics of the output light of a Raman fiber laser with feedback in the laminar-turbulent transition. By using an ordinal patterns approach, we have found signatures of determinism in its dynamics that allow us to forecast high-intensity events, defined as events larger than a threshold ($th^{*}>2\sigma$). The system presents preferred patterns before it changes from low-intensity events to more extreme events. The preferred pattern before an extreme event is the one where the latter of three consecutive low-intensity events is larger than the previous two, with probabilities that reach 80% in some cases, while the less probable pattern is the one where the latter event is less intense than the previous two events, which in some cases reaches less than 10%.

We can compute the patterns with the intensity of the events or with the inter-event time-intervals. The former takes advantage of intensity correlations in the system, while the latter of temporal correlations, both being present in the dynamics. In this system, the more efficient way to compute the patterns for forecasting purposes is with the actual intensity of the events. For patterns computed with inter-event time-intervals, the probabilities, although still not compatible with a stochastic process, are lower and therefore less efficient to forecast the more extreme events.

The fact that there is 70% higher probability of pattern XX2 before a sudden increase in intensity has to be considered carefully, as there are much fewer events that are higher than $th^{*}$ than events below $th_{0}$. This does not mean that 70% of all XX2 patterns will be followed by a high-intensity event, but that 70% of high-intensity events are preceded by pattern XX2.

One interpretation for this preference for specific patterns when the system leaves the low-intensity regime and explores the higher intensity regime is that there is a specific region in phase space that triggers extreme events, and those patterns are the signature of that region in phase space.

Our analysis covered the laminar-turbulent transition in the Raman fiber laser. In a previous work, the OPAM had been shown to be an efficient tool to characterize this transition, and a sharp decrease of entropy was found right at the transition, with respect to the well-developed laminar or turbulent regimes. Our current work has found that, despite having clear, preferred forecasting patterns, these are the same all through the pump current range (laminar, transition, and turbulent). This suggests that the temporal and intensity correlations found are a signature of the structure of the phase space and not related to the particular features of each of the regimes.

We have also found that the forecasting signatures of the system are higher in the turbulent regime than in the laminar regime. The more complex dynamics of the turbulent regime facilitates the appearance of structures in the dynamics that help identify sharp changes from low-intensity to high-intensity events.

This work is also in agreement with the previous work, where also the hierarchy of ordinal patterns found did not change. However, the fact that the preferred patterns in both cases are different (all the patterns in the time series on the one hand versus those forecasting a low-high event change) indicates that the system visits a different region of phase space when a change from a low-intensity event to a high-intensity event is happening.

This methodology can be applied to other complex dynamical systems that manifest time series of events, with a long-tail histogram.
